# Impact of inducing general anesthesia with Propiscin (etomidate) on the physiology and health of European perch (*Perca fluviatilis* L.)

**DOI:** 10.1007/s10695-018-0482-4

**Published:** 2018-02-23

**Authors:** Maciej Rożyński, Krystyna Demska-Zakęś, Agnieszka Sikora, Zdzisław Zakęś

**Affiliations:** 1 Department of Aquaculture, The Stanisław Sakowicz Inland Fisheries Institute, Oczapowskiego 10, 10-719 Olsztyn, Poland; 2 Department of Ichthyology, Faculty of Environmental Sciences, University of Warmia and Mazury, Oczapowskiego 5, 10-719 Olsztyn, Poland

**Keywords:** Analgesia, Anesthetic, Biochemical blood indicators, Hematology, European perch

## Abstract

The aim of the study was to describe the course and timing of the different stages of anesthesia induced with Propiscin (etomidate) on juvenile European perch (experiment I) and to describe the effect of immersing specimens of this species had on selected hematological and biochemical parameters (experiment II). The study was conducted on material with body weights (BW) of 162.98 (experiment I) and 171.60 g (experiment II). In experiment I, general anesthesia was induced with two different anesthetic concentrations (1 or 2 ml l^−1^; anesthesia time 10 min). In experiment II, blood was drawn for hematological and biochemical analyses from the fish that had been exposed to anesthetic immersion baths with two different concentrations of Propiscin (1 and 2 ml l^−1^) and for different exposure times (3 and 10 min). Blood samples were collected immediately following immersion (0 h) and 24 h later (24 h). Specimens that were immersed at the higher concentration of anesthetic achieved subsequent stages of general anesthesia two times faster (*P* ≤ 0.05). However, during recovery, some statistically significant differences were observed, but these lasted only until stage I was achieved. Among the hematological parameters (0 h), significant differences were observed in hematocrit (HCT) and mean corpuscular volume (MCV), while among the biochemical determinations (0 h), statistically significant differences were noted in the concentrations of glucose, calcium, lactate, and ammonia. After 24 h, the levels of these parameters in all fish groups returned to initial values. The hematological and biochemical tests conducted permit concluding that the anesthetic tested, at the concentrations (1 and 2 ml l^−1^) and the exposure times of up to 10 min at which it was tested, is safe and can be used successfully to induce general anesthesia in perch.

## Introduction

Percid fishes are among the so-called promising species for aquaculture (Kestemont et al. 2015a). In Europe over the past two decades, particular attention in aquaculture has been focused on two species: pikeperch (*Sander lucioperca*) and European perch (*Perca fluviatilis*). In the case of the second of the two taxa, fairly precise biological techniques have been developed for culturing larval and juvenile stages and for ongrowing in various culture systems (Toner and Rougeot 2008, Kestemont et al. 2015b, Policar et al. 2015). In the context of intensifying perch production, the most promising technique appears to be culturing this species in recirculating aquaculture system (RAS) (Kestemont et al. 2015b). Intense production in RAS requires frequent handling (i.e., controlled spawning, tagging, transporting, sorting, taking monitoring measurements, stocking tanks). All of these stress-inducing procedures can negatively impact the health status of fish (Falahatkar and Barton 2007). Anesthetics are used to reduce the impact of stress on fish, and the use of them in aquaculture is becoming increasingly common (Husen and Sharma 2014). Anesthetics are used with percids, for example, to separate larval individuals with inflated swim bladders from those with uninflated swim bladders (Kestemont et al. 2015b), and they are also used when collecting sex products during controlled spawning (Zakęś et al. 2013; Alavi et al. 2015).

Various anesthetics have been tested to induce general anesthesia in perch, including Propiscin (*Etomidatum*, IFI (Inland Fisheries Institute), Olsztyn), MS-222 (*Tricaine methanesulfonate*, Sigma-Aldrich, St. Louis, MO, USA), clove oil (*Oleum Caryophyllorum*), and 2-phenoxyethanol (*Phenoxyethanolum*). Among the anesthetics tested to date on perch, MS-222 and Propiscin are recommended (Hamáčková et al. 2001; Velisek et al. 2009). Propiscin is a stable 0.2% etomidate solution (Kazuń and Siwicki 2012) that is effective in inducing anesthesia in fish species such as rainbow trout (*Oncorhynchus mykiss*), carp (*Cyprinus carpio*), grass carp (*Cteropharyngodon idella*), and grayling (*Thymallus thymallus*) (Kazuń and Siwicki 2012; Witeska et al. 2015). Propiscin has been tested on perch by Hamáčková et al. (2001) and Velisek et al. (2009); however, these studies did not provide a complete picture of the impact this anesthetic has on the bodies of the fish. The Propiscin concentration applied most commonly is 1.5–2 ml l^−1^, but the only concentration tested in the studies mentioned above was 1 ml l^−1^. Additionally, these studies do not describe the effect this anesthetic has on the hematological profile, while the described effect that using Propiscin has on the biochemical profile is incomplete. Assessing hematological and biochemical parameters is a key tool in assessing the health and condition vertebrates, which includes fish (Collins et al. 2016). Performing a complete blood count is a basic diagnostic procedure. Assessing the levels and ranges of, inter alia, hemoglobin (HGB), hematocrit (HCT), erythrocytes, leukocytes, and thrombocytes permits detecting anemia, inflammation, infection, or other disease processes (Clauss et al. 2008). Complete blood counts are also often used in toxicity studies of various substances (Javed and Usmani 2015). Biochemical parameters are also very useful in assessing the physiological state of living organisms, and they facilitate monitoring the functioning of many organs (e.g., liver, kidney, heart) and glands, nutritional status, or systemic hydration (e.g., total protein (TP), magnesium (Mg), calcium (Ca)), and the occurrence of the stress phenomenon (e.g., glucose (GLU), lactate (LACT)) (Haluzova et al. 2010; Brinn et al. 2012; Rożyński et al. 2017).

The aim of the study was to describe the course and timing of the various stages of anesthesia induced with Propiscin (etomidate) in juvenile perch and to determine the impact that immersing specimens of this species in an aqueous solution of anesthetic had on selected hematological and biochemical indicators.

## Materials and methods

### Fish—origin, rearing conditions

The study material was obtained by collecting fertilized perch eggs from spawning grounds during the natural spawning period of this species (early April) in Lake Dgał Wielki (Masurian Lake District, northeastern Poland). The eggs were placed in an earthen pond with a surface area of 0.2 ha (Department of Sturgeon Fish Breeding, Inland Fisheries Institute in Olsztyn, northeastern Poland (IFI Olsztyn)). The fish were reared in the pond to the summer fry stage (body weight (BW) approximately 0.2 g)) on natural food. In mid-June, the fry were collected from the pond and placed in a RAS (two tanks with a cubic volume of 2 m^3^). After the fry were moved to the RAS, they were trained to consume formulated feed (Nutra, Nutreco, Trouvit, France). The feeding regime applied during the initial rearing period was based on principles described by Policar et al. (2015). When the fry had attained a BW of 10 g, approximately 500 specimens were transported (polyethylene bags, 20-l water + 20-l oxygen, transport time—2 h) to the Department of Aquaculture (IFI Olsztyn). The fish were stocked into culture tanks in a RAS, which was an experimental setup equipped with, inter alia, rearing tanks with a volume of 200 l and biological filters containing RK BioElements (RK Plast, Denmark) (Z. Zakęś, unpublished materials). During rearing in the RAS, optimal physical and chemical rearing parameters were maintained: water temperature—19.7 ± 0.1 °C; pH range—7.80–8.01; oxygenation at the rearing tank outflows did not decrease below 7.3 mg O_2_ l^−1^; the total ammonia nitrogen concentration (TAN = NH_4_^+^-N + NH_3_-N) measured at the rearing tank outflows did not exceed 0.2 mg TAN l^−1^, and that of nitrites (NO_2_-N) did not exceed 0.1 mg NO_2_-N l^−1^. The fish were fed with an automatic band feeder (Fischtechnik GmbH, Nienburg, Germany), and the feed was delivered continually for 19 h day^−1^ (10:00–05:00). During the rearing period, the fish were fed T-T Nutra MP feed (Skretting, the Netherlands) with a composition of protein—52%, crude fat—20%, carbohydrate—9.5%, crude fiber—0.5%, and an energy value of 19.9 MJ/kg.

### Experimental procedure

Two experiments were conducted. Experiment I focused on determining the time required to induce general anesthesia and the different stages of general anesthesia during immersion in an aqueous solution of Propiscin (etomidate) at two different concentrations (1 and 2 ml 1^−1^). The procedure used was that described by Hamáčková et al. (2001) and Kazuń and Siwicki (2001). The fish used in the study had a mean BW of 162.98 ± 29.40 g and a body length (SL) of 20.8 ± 1.2 cm. They were divided into two groups of ten specimens each (*n* = 10). All specimens were immersed for 10 min in an immersion bath containing an aqueous solution of Propiscin, but each group was exposed to different concentrations (group PROP1—1 ml l^−1^, group PROP2—2 ml l^−1^) of the anesthetic. Immersion was conducted in 20-l containers filled with the etomidate solution mixed with water from the RAS collected at the outflows of the rearing tanks. The temperature of the immersion bath was similar to that maintained in the RAS throughout the rearing period. During the induction of general anesthesia, a stopwatch was used to time how long each fish took to reach subsequent stages of anesthesia (± 0.1 s; Table 1). After 10 min, the anesthetized fish were moved to a tank of fresh, oxygenated water from the RAS. Next, the course of recovery and the time required to reach subsequent stages of recovery from general anesthesia were observed (Table 1). The fish were immersed individually after the preceding specimen had fully recovered.

**Table 1:** Description of the different phases of the induction of and recovery from general anesthesia in fish (after Hamáčková et al. 2001; Kazuń and Siwicki 2001)

Activity	Phase	Phase description
Anesthesia	Phase I Quiet behavior	Physiological position Regular respiratory motion Normal locomotor activity Effortless evading obstacles when swimming
	Phase II Excitation	Physiological position Restlessness Fast swimming Not evading the obstacles when swimming Strong withdrawal reflex Shallow, irregular respiratory motion
	Phase IIIA Total anesthesia (superficial)	Slight tilting on the flank Decreased activity Weakened or no withdrawal reflex Respiratory motions regular, slower and deep
	Phase IIIB Total anesthesia (complete)	Flank position Loss of motility None of the withdrawal reflexes but the acoustic one Respiratory motions regular, deep, retarding
	Phase IV Respiration block	Flank position Respiratory motions blocked or superficial to involuting No withdrawal reflex, neither the acoustic one
Anesthesia recovery	Phase I	Flank position Regular respiration Acoustic reflex
	Phase II	Flank position changed to physiological one Uncoordinated motions Regular respiration
	Phase III	Physiological position Slow swimming initiated Uncoordinated motions Not evading the obstacles when swimming
	Phase IV	Physiological position Normal locomotor activity Normal swimming Evading the obstacles when swimming

In experiment II, the effect of immersion in the aqueous solution of Propiscin at two different concentrations (1 and 2 ml 1^−1^) and for two different periods (3 and 10 min) on the hematological and biochemical indicators of perch blood (BW = 171.60 ± 39.00 g; SL = 21.9 ± 1.4 cm) was determined. The fish were divided into nine groups (eight experimental groups and one control group) of seven specimens each (*n* = 7) (Table 2). Immersion was conducted in 20-l containers filled with the etomidate solutions mixed with water from the RAS with the following physical and chemical parameters: water temperature—21.4 °C, oxygen concentration—8.31 mg O_2_ l^−1^, water pH—8.11, electrolytic conductivity—446 μS cm^−1^, and general hardness—295.9 CaCO_3_ l^−1^. The methodology for immersing and collecting blood samples was similar to that described by Velisek et al. (2009, 2011). Blood samples (approximately 2 ml) were drawn from each fish specimen from four groups immediately after immersion ended (0 h). All manipulations were completed with each fish specimen before the procedure was begun with the subsequent specimen. However, with the four subsequent groups, for which the immersion regime was identical, immersion was conducted in groups under similar conditions. Next, each of these groups was placed in a separate tank in the RAS. Blood was drawn from the fish in these groups 24 h after the conclusion of immersion (Table 2). The control group comprised specimens that were immersed in RAS water (without anesthetic) in a container of the same volume as was used in the experimental groups. Blood was drawn from all specimens with pre-heparinized syringes (Smiths Medical, Minneapolis, USA) from the tail vein. After collection, the blood was analyzed in a semi-automatic BC2800Vet hematological apparatus (Mindray, Shenzhen, China). Some of the blood samples were centrifuged (4000 rpm; 20 °C; 3 min; Fresco 17, Thermo Scientific, Waltham, USA) and subjected to biochemical analysis in an automatic BS120 apparatus (Mindray, Shenzhen, China). The most important hematological parameters were selected for analysis, as follows: white blood cell count (WBC), red blood cell count (RBC), platelet count (PLT), HGB, HCT, mean corpuscular volume (MCV), mean corpuscular hemoglobin (MCH), and mean corpuscular hemoglobin concentration (MCHC). The following biochemical parameters were determined as follows: creatinine (CREA), TP, total bilirubin (BIL-T), alanine aminotransferase (ALT), alkaline phosphatase (ALP), aspartate aminotransferase (AST), albumin (ALB), globulin (GLB), GLU, Ca, Mg, chloride (Cl), LACT, ammonia (NH_3_).

**Table 2:** Experiment setup for the application of the anesthetic (Propiscin) to perch (*n* = 7)

Specification	Units	Control group	Experimental groups							
			1	2	3	4	5	6	7	8
Concentration	ml l^−1^	0	1	1	2	2	1	1	2	2
Exposure time	min	0	3	10	3	10	3	10	3	10
Sampling	h	0	0	0	0	0	24	24	24	24

### Statistical analysis

The results were analyzed statistically with the Statistica 12 computer program (StatSoft, Inc., USA). The data were tested for normality of distribution (Shapiro-Wilk’s *W* test) and homogeneity of variance (Levene’s test). Statistical comparisons of data from stage I were performed with Student’s *t* test for independent samples, while the data from stage II were compared with one-way analysis of variance (ANOVA). When statistical significance was confirmed, further statistical analysis was conducted with Tukey’s test. Differences were significant at *P* ≤ 0.05.

## Results

**Fig. 1:**
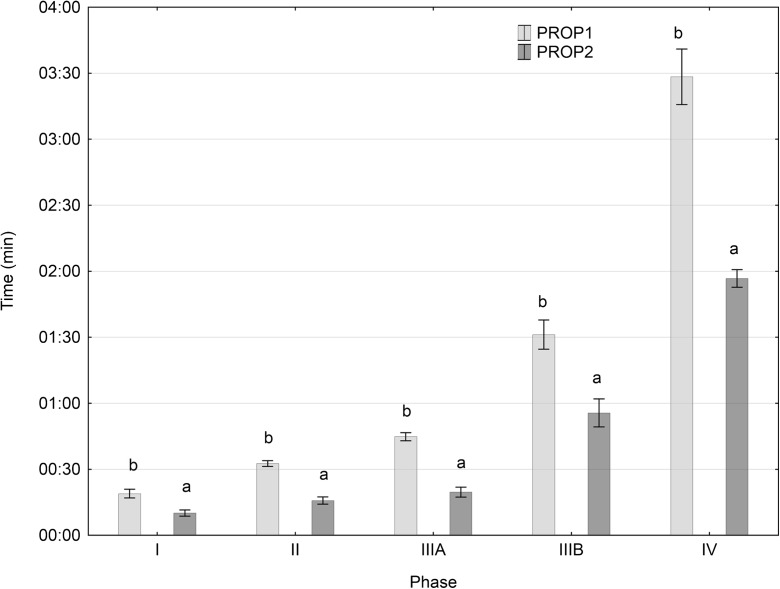
Impact of Propiscin concentration on the induction of general anesthesia in perch. Groups marked with a letter index within a given stage differ significantly statistically (*P* ≤ 0.05) (mean values ± SE)

**Fig. 2:**
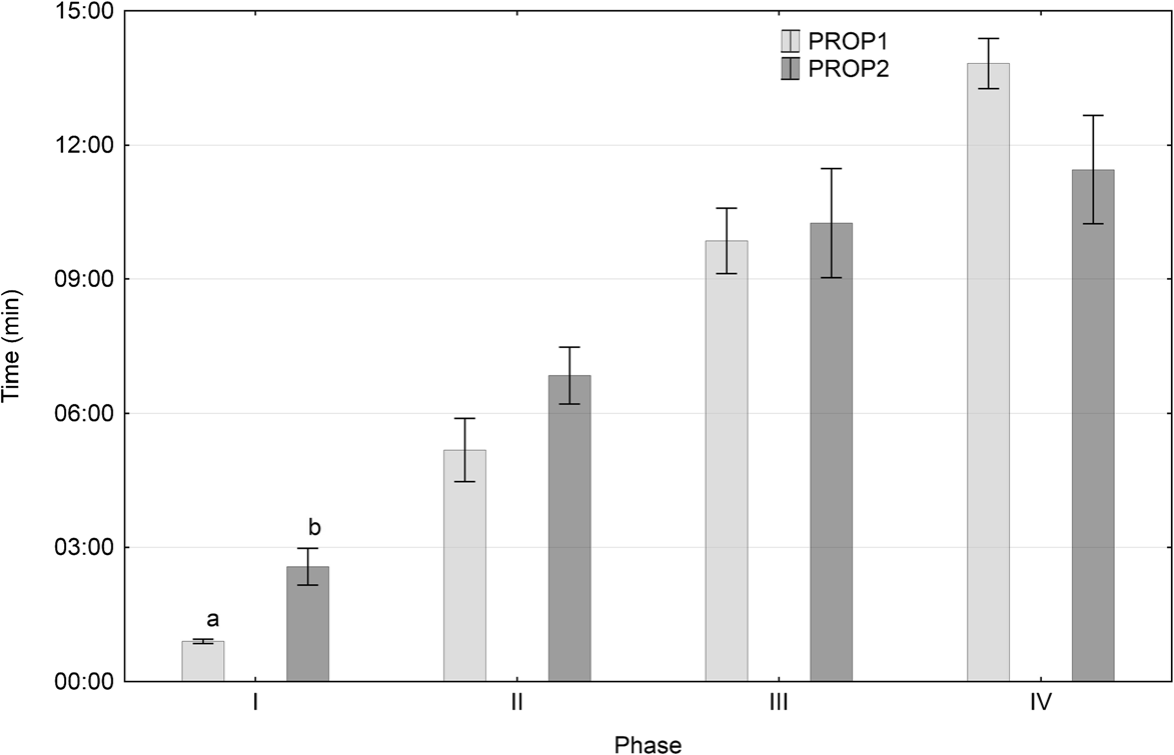
Impact of Propiscin concentration on the recovery from general anesthesia in perch. Groups marked with a letter index within a given stage differ significantly statistically (*P* ≤ 0.05) (mean values ± SE)

Immersion in the higher concentration of Propiscin (2 ml l^−1^) permitted inducing general anesthesia approximately twice as fast as with the 1-ml l^−1^ solution. The same was observed regarding the different stages of general anesthesia. The time required for the fish immersed in the higher anesthetic concentration to reach the subsequent stages of anesthesia was twice as short, and the differences observed were statistically significant (*P* ≤ 0.05; Fig. 1) in comparison to group PROP1. During the recovery of the fish from general anesthesia, the opposite was observed. Only during stage I of recovery from general anesthesia was the difference noted between the recovery times of the two groups significant. In group PROP2, the time to attain stage I was approximately three times longer (*P* ≤ 0.05; Fig. 2). However, the time required to achieve the subsequent stages (stages II–IV) was very similar in both groups and did not differ significantly (*P* > 0.05; Fig. 2).

Among all the hematological indicators determined, significant differences were noted only immediately following immersion (0 h) and only in the case of two of the parameters. Significant increases in HCT (of approximately 19.5%) in comparison to the control groups were noted in the fish immersed in the Propiscin solution at a concentration of 1 ml l^−1^ for 10 min (*P* ≤ 0.05; Table 3). In all experimental groups, elevated MCV levels were noted immediately after the procedure concluded (0 h). Statistically significant differences among groups were confirmed only in three of them (2 ml l^−1^/3 min; 1 ml l^−1^/10 min; 2 ml l^−1^/10 min; *P* ≤ 0.05; Table 3). After 24 h, the values of both parameters in all experimental groups were comparable with those of the control group.

**Table 3:** Impact of general anesthesia induced by immersion in a Propiscin bath (anesthetic concentration of 1 or 2 ml l^−1^/immersion time 3 or 10 min) on the hematological profile of perch blood (mean value (± SE)) (*n* = 7). Details are provided in “Materials and methods”

Specification	Units	Control group	Time (0 h)				Time (24 h)			
			1 ml/3 min	2 ml/3 min	1 ml/10 min	2 ml/10 min	1 ml/3 min	2 ml/3 min	1 ml/10 min	2 ml/10 min
WBC	10^3^ μl^−1^	95.76 (± 3.67)	107.78 (± 3.33)	121.09 (± 4.09)	114.07 (± 6.53)	128.08 (± 11.83)	113.93 (± 11.41)	112.31 (± 3.88)	103.39 (± 7.62)	118.33 (± 6.18)
RBC	10^6^ μl^−1^	1.47 (± 0.07)	1.64 (± 0.10)	1.58 (± 0.06)	1.61 (± 0.07)	1.61 (± 0.10)	1.52 (± 0.12)	1.62 (± 0.04)	1.53 (± 0.09)	1.65 (± 0.06)
HGB	g l^−1^	28.06 (± 1.40)	31.28 (± 1.43)	33.46 (± 1.07)	31.11 (± 1.52)	30.30 (± 2.05)	28.67 (± 1.92)	33.55 (± 1.68)	28.58 (± 1.45)	30.41 (± 2.02)
HCT	%	29.30 (± 1.35)^a^	34.06 (± 1.64)^ab^	35.74 (± 1.13)^ab^	36.39 (± 1.09)^b^	36.05 (± 1.91)^ab^	29.70 (± 1.91)^ab^	33.60 (± 1.10)^ab^	30.49 (± 1.58)^ab^	32.54 (± 1.44)^ab^
MCV	fl	148.24 (± 2.48)^a^	154.67 (± 3.19)^ab^	168.23 (± 1.92)^b^	168.89 (± 3.46)^b^	168.10 (± 5.03)^b^	146.13 (± 5.02)^a^	154.04 (± 1.89)^ab^	148.31 (± 1.84)^a^	147.23 (± 6.11)^a^
MCH	pg	31.34 (± 1.07)	31.40 (± 1.08)	34.87 (± 1.39)	31.94 (± 1.66)	31.50 (± 2.25)	31.31 (± 1.93)	33.86 (± 1.02)	30.71 (± 0.76)	30.36 (± 2.07)
MCHC	g l^−1^	211.71 (± 4.95)	203.14 (± 3.48)	207.29 (± 5.63)	188.57 (± 6.85)	186.00 (± 8.18)	213.29 (± 6.10)	219.86 (± 4.1)	207.14 (± 3.46)	205.43 (± 6.34)
PLT	10^3^ μl^−1^	16.57 (± 3.32)	19.57 (± 1.81)	19.71 (± 2.35)	19.43 (± 3.95)	19.67 (± 2.7)	40.86 (± 24.69)	17.86 (± 3.35)	16.00 (± 0.76)	22.29 (± 1.90)

Groups in rows marked with a different letter index differ significantly statistically (*P* ≤ 0.05)

The analysis of the biochemical indicators of blood plasma conducted immediately following the conclusion of immersion (0 h) showed statistically significant differences in five of the 14 parameters determined (Table 4). The highest increase in peripheral blood GLU levels in the fish examined was confirmed in the group subjected to the 3 min immersion at the lowest concentration of Propiscin (1 ml l^−1^). The value of this parameter was over twice as high as that in the control groups and it differed statistically significantly from the other three experimental groups. Increased GLU levels were also confirmed in the group of fish anesthetized at the higher anesthetic concentration for the same period of time (2 ml l^−1^/3 min) (*P* ≤ 0.05; Table 4). The concentrations of GLU of the fish subjected to longer immersion times of 10 min and at both anesthetic concentrations (1 or 2 ml l^−1^) were higher, but these differences were not significant in comparison to those of the control groups (*P* > 0.05). Twenty-four hours after the conclusion of immersion, the concentration of GLU in the fish from all groups was similar to that in the control groups (*P* > 0.05; Table 4). At 0 h in all experimental groups, elevated blood Ca concentrations were noted; however, only in the group subjected to immersion at the lowest anesthetic concentration and for the shortest period of time (1 ml l^−1^/3 min) were these differences significant (*P* ≤ 0.05; Table 4), and after 24 h, the concentration of this ion returned to initial levels. No differences in blood Mg concentrations were noted immediately following the conclusion of immersion (0 h) among the various groups; however, after 24 h, a significant decrease was noted in the level of this electrolyte in the peripheral blood in two groups that were immersed in the lower concentration of anesthetic (1 ml l^−1^/3 or 10 min) in comparison to that in the control groups (*P* ≤ 0.05; Table 4). Significant increases in LACT concentration values in perch peripheral blood immediately following the conclusion of immersion (0 h) were confirmed in the groups exposed to the anesthetic for 3 min at both concentrations tested (1 and 2 ml l^−1^) and also after the application of the anesthetic at a concentration of 1 ml l^−1^ for 10 min (Table 4). After 24 h, the value of this parameter in all groups was comparable to the initial value. Higher NH_3_ values were observed in all four experimental groups (0 h); however, significantly higher concentrations of NH_3_ (approximately twice as high as that in the control groups) were confirmed only in the group subjected to immersion in the highest anesthetic concentration and for the shortest period (2 ml l^−1^/3 min). After 24 h, the concentration of NH_3_ in this group did not differ from that noted in the remaining groups (Table 4).

**Table 4:** Impact of general anesthesia induced by immersion in a Propiscin bath (anesthetic concentration 1 or 2 ml l^−1^/immersion time 3 or 10 min) on the biochemical profile of perch blood (mean value (± SE)) (*n* = 7). Details are provided in “Materials and methods”

Specification	Units	Control group	Time (0 h)				Time (24 h)			
			1 ml/3 min	2 ml/3 min	1 ml/10 min	2 ml/10 min	1 ml/3 min	2 ml/3 min	1 ml/10 min	2 ml/10 min
BIL-T	mg dl^−1^	0.11 (± 0.04)	0.11 (± 0.01)	0.11 (± 0.01)	0.18 (± 0.05)	0.18 (± 0.04)	0.09 (± 0.01)	0.11 (± 0.02)	0.09 (± 0.01)	0.16 (± 0.03)
ALT	U l^−1^	24.00 (± 8.14)	14.20 (± 4.53)	21.86 (± 6.49)	19.71 (± 3.28)	26.71 (± 8.40)	15.71 (± 5.06)	22.00 (± 5.2)	8.86 (± 2.34)	9.14 (± 2.55)
ALP	U l^−1^	39.86 (± 3.38)	49.00 (± 2.16)	44.71 (± 4.60)	45.14 (± 2.60)	43.00 (± 5.54)	46.00 (± 5.67)	43.33 (± 5.15)	36.29 (± 3.56)	42.71 (± 2.00)
AST	U l^−1^	175.57 (± 43.05)	114.20 (± 15.18)	131.71 (± 42.24)	142.29 (± 22.81)	165.43 (± 54.86)	103.43 (± 37.72)	183.00 (± 35.28)	57.00 (± 16.22)	95.00 (± 20.96)
TP	g dl^−1^	4.03 (± 0.20)	4.54 (± 0.12)	4.60 (± 0.27)	4.24 (± 0.20)	4.27 (± 0.30)	3.83 (± 0.31)	4.49 (± 0.16)	3.98 (± 0.14)	4.26 (± 0.14)
ALB	g dl^−1^	1.40 (± 0.06)	1.55 (± 0.02)	1.52 (± 0.05)	1.51 (± 0.05)	1.47 (± 0.08)	1.42 (± 0.11)	1.61 (± 0.09)	1.46 (± 0.04)	1.56 (± 0.04)
GLB	g dl^−1^	2.64 (± 0.14)	2.99 (± 0.10)	3.08 (± 0.22)	2.72 (± 0.17)	2.80 (± 0.23)	2.41 (± 0.21)	2.87 (± 0.07)	2.52 (± 0.10)	2.71 (± 0.10)
GLU	mg dl^−1^	100.00 (± 8.91)^ac^	229.20 (± 14.37)^d^	156.43 (± 9.22)^b^	125.00 (± 7.24)^bc^	116.86 (± 7.94)^ab^	82.71 (± 8.97)^a^	89.50 (± 3.64)^ac^	79.14 (± 2.47)^a^	100.57 (± 11.74)^ac^
Ca	mg dl^−1^	10.52 (± 0.27)^a^	12.30 (± 0.23)^b^	12.17 (± 0.35)^ab^	11.09 (± 0.43)^ab^	11.66 (± 0.24)^ab^	9.80 (± 0.78)^a^	10.90 (± 0.16)^ab^	10.44 (± 0.18)^ab^	11.31 (± 0.35)^ab^
Mg	mg dl^−1^	2.88 (± 0.13)^b^	2.95 (± 0.04)^b^	3.10 (± 0.10)^b^	2.91 (± 0.08)^b^	2.96 (± 0.12)^b^	2.37 (± 0.17)^a^	2.69 (± 0.15)^ab^	2.37 (± 0.03)^a^	3.03 (± 0.07)^b^
Cl^−^	mmol/l	243.27 (± 14.83)^ab^	273.80 (± 10.78)^b^	270.53 (± 11.24)^b^	246.34 (± 14.04)^ab^	259.67 (± 11.37)^ab^	207.27 (± 21.99)^a^	249.65 (± 7.21)^ab^	225.49 (± 7.18)^ab^	249.74 (± 14.98)^ab^
LACT	mg dl^−1^	6.59 (± 1.28)^ab^	29.36 (± 0.40)^d^	27.61 (± 0.65)^d^	20.71 (± 1.25)^cd^	16.96 (± 1.17)^abd^	7.54 (± 1.71)^ac^	3.65 (± 0.24)^a^	3.74 (± 0.56)^a^	18.33 (± 8.39)^bcd^
CREA	mg dl^−1^	0.45 (± 0.06)^ac^	0.22 (± 0.03)^a^	0.34 (± 0.05)^ab^	0.53 (± 0.09)^bc^	0.63 (± 0.08)^c^	0.43 (± 0.08)^ac^	0.50 (± 0.06)^ac^	0.29 (± 0.03)^a^	0.40 (± 0.03)^ac^
NH_3_	μg dl^−1^	78.83 (± 5.84)^ac^	104.74 (± 6.20)^cd^	143.49 (± 11.66)^d^	109.43 (± 14.05)^cd^	102.83 (± 6.59)^c^	70.91 (± 8.27)^ac^	78.02 (± 8.67)^ac^	59.46 (± 2.39)^a^	81.23 (± 10.67)^ac^

Groups in rows marked with a different letter index differ significantly statistically (*P* ≤ 0.05)

## Discussion

Using anesthetics in aquaculture facilitates handling fish faster and more efficiently while also minimizing the magnitude of losses that follow handling (Weber et al. 2009; Kazuń and Siwicki 2012). In the current study, both concentrations of Propiscin tested (1 and 2 ml l^−1^) permitted inducing general anesthesia in perch in a period of time that was close to the optimum for inducing anesthesia in fish, which is approximately 3 min (Park et al. 2008). Data available in the literature regarding the induction of general anesthesia in Percidae indicate that achieving this in species of this family using Propiscin can last from 49 s (pikeperch; 1.5 ml l^−1^; 23 °C; BW = 7.88 g) to even approximately 5 min (perch; 1 ml l^−1^; 20 °C; BW = 32.20 g) (Hamáčková et al. 2001; Kristan et al. 2014). This discrepancy can be explained by the dependence between the time it takes to induce general anesthesia in fish and many factors, from the concentration of anesthetic applied, to the physicochemical parameters of the water, and the species and size of the fish being anesthetized (Son et al. 2001). In the current study, the recovery of perch from general anesthesia, in contrast to its induction, was not correlated with the concentration of Propiscin applied.

Regardless of the anesthetic used, its concentration, or the physicochemical parameters of the environment, inducing general anesthesia in fish causes them to suffer from the stress phenomenon (Simon et al. 1983). The changes in peripheral blood parameters in perch observed in this study suggest that immersing them in an aqueous solution of Propiscin causes minor stress. As a result of the body’s elevated oxygen requirements, the amount of HGB increases in the erythrocytes to increase the transport of oxygen molecules to different tissues and organs. Evidence of this is seen in the elevated values of MCV and HCT and increased levels of HGB, although these were insignificant (Rutten et al. 1992). Stress in perch only occurred during immersion in anesthetic, because 24 h after the conclusion of this procedure in all groups, these parameters were noted to have returned to initial values. Similar reactions to immersion in an aqueous solution of etomidate (Propiscin; 1.5 ml l^−1^; 10 min; 20.5–20.7 °C) were not observed in juvenile pikeperch (BW = 71.48 g). Nevertheless, significantly higher values of mean corpuscular HGB concentrations were noted both immediately following immersion and 24 h after it (Kristan et al. 2012). In perch, similarly to the other hematological indicators, they were comparable to those confirmed in the control group (this study).

Biochemical blood tests are good indicators of anesthetic quality, since they deliver much valuable information regarding the physiological state of animals and the occurrence of stress in their bodies (Barcellos et al. 2003). One important biochemical indicator is GLU. Increased levels of this monosaccharide in perch peripheral blood is probably a consequence of the release into the blood of catecholamines (inter alia, adrenaline) and cortisol that accompany the impact that stressors (in this case, an anesthetic) have on bodies (Simon et al. 1983). Comparing the results obtained from different groups of perch studied, one can conclude that only the first few minutes of immersion in Propiscin impacts blood GLU levels. After this period, the concentration of this compound in perch peripheral blood decreases. Analyzing the results of blood GLU levels in two groups of fish that were subjected to immersion for 3 min, one can conclude that the faster general anesthesia is induced, the lower the level of stress experienced by the fish is. Among juvenile pikeperch (BW = 189.89 g) that were anesthetized in an aqueous solution of etomidate (Propiscin; 1 or 2 ml l^−1^/2 or 10 min; 21.3 °C), elevated GLU levels were observed only after 10 min of immersion, and this was regardless of the anesthetic concentration applied. However, in this study as well, 24 h later, the level of this saccharide in the peripheral blood of the studied fish had returned to the initial values (Rożyński et al. 2016). However, among adult vimba bream (*Vimba vimba*) (BW = 339.21 g), which were placed under general anesthesia using Propiscin (1 ml l^−1^; 10 min; 17.6–18.2 °C), elevated blood GLU levels were observed to persist even longer than 24 h after the conclusion of immersion (Lepic et al. 2014).

Elevated LACT concentrations in the peripheral blood were observed in perch immediately after immersion in Propiscin ended, which also indicates the occurrence of the phenomenon of stress (Mushtaq et al. 2014). Stress leads to increased energy expenditures in the body and increases oxygen requirements substantially. Since the oxygen delivery system (erythrocytes) is often unable to meet this demand, glycolysis occurs in cells under anaerobic conditions, which results in the release of lactic acid from the muscles. Comparing our findings to those from studies of other fish, one can conclude that the perch response to the induction of general anesthesia with Propiscin is conditioned by factors that are particular to this species. For example, this parameter was not noted to have increased in juvenile pikeperch (BW = 71.48 g) following immersion in a solution of etomidate (Propiscin; 1.5 ml l^−1^/10 min; 20.5–20.7 °C), while after rainbow trout had been immersed a decrease in the level of LACT in the peripheral blood was noted (Velisek et al. 2011; Kristan et al. 2012). The analysis of Mg levels in the blood of perch also permits concluding that the induction of general anesthesia in this species causes a stress reaction. Mg plays a number of important functions in the body such as participating in GLU transport, producing energy, and impacting nerve impulse transmission and the muscles (contracting and relaxing) (Bijvelds et al. 1998). As was previously mentioned, the stress hormones adrenalin and cortisol stimulate the body, increase energy demand, and trigger a number of enzymatic and biochemical reactions. Mg plays a role in many of these, which, consequently, can lead to deficits and even depletion of this ion. This phenomenon of decreased Mg was noted in perch 24 h after the conclusion of immersion in Propiscin (especially at the concentration of 1 ml l^−1^). However, immediately after exposure (0 h), the concentration of this electrolyte was comparable to that of the control group. In pikeperch (BW = 189.89 g), elevated blood levels of this ion were noted immediately after immersion (0 h) in the anesthetic solution regardless of its concentration or the length of the immersion (Rożyński et al. 2016). Nevertheless, after 24 h, these levels returned to values that were comparable to the control group. Kristan et al. (2012) report yet another situation in juvenile pikeperch (BW = 71.48 g) noting that there was no significant change in the concentration of this electrolyte during general anesthesia induced with Propiscin. The opposite reaction described above (blood Mg concentration) to the induction of general anesthesia using Propiscin in those fish probably stemmed from the different sizes of the fish used in the study.

In one of the perch groups (1 ml l^−1^/3 min), elevated Ca ions were noted immediately after immersion (0 h). Rożyński et al. (2016) observed a similar phenomenon in all groups of pikeperch (BW = 189.89 g) they studied; however, in the studies of both pikeperch and perch, Ca levels retuned to initial levels after 24 h. However, Kristan et al. (2012) did not observe any significant changes in this parameter during the induction of general anesthesia in pikeperch (BW = 71.48 g) using Propiscin. Elevated levels of NH_3_ in perch observed in the current study immediately after the conclusion of immersion (0 h) could have been caused by augmented protein catabolism or disruptions in the excretion of this metabolite from the system (Svoboda 2001). Similar observations of elevated NH_3_ levels immediately following the conclusion of immersion were reported for juvenile pikeperch with BW = 189.89 g (Rożyński et al. 2016). In turn, slight decreases in NH_3_ levels were noted in smaller pikeperch (BW = 71.48 g) immediately following the completion of immersion, but 24 h later, these levels were more than three times lower than those in the control groups (Kristan et al. 2012). Higher peripheral blood NH_3_ concentrations were also confirmed in vimba bream (BW = 339.21 g); however, observations of this species were not done until 24 h after the completion of immersion in Propiscin (Lepic et al. 2014). In turn, Velisek et al. (2009) did not note any changes at all in NH_3_ concentrations in perch (BW = 47.20 g) during the induction of general anesthesia in an aqueous solution of Propiscin (1 ml l^−1^/10 min; 20.0–20.2 °C). The information above indicates that the reaction of fish to a given anesthetic exhibits not only a great degree of species specificity, but that reactions can also be specific to given ontogenetic developmental stages.

## Conclusions

Taking into consideration the anesthesia recovery rates of the fish and recovery times, the low intensity of stress reactions, and the 100% fish survival rate following the procedure, it is justified to recommend using a Propiscin solution at a concentration of 2 ml l^−1^ during rearing procedures that require anesthetizing this species. Additionally, the hematological and biochemical tests performed also permit concluding that the anesthetic tested at the concentrations tested (1 and 2 ml l^−1^) is safe and can be used successfully to induce general anesthesia.
